# Haploidentical Hematopoietic Stem-Cell Transplantation in Adults

**DOI:** 10.1155/2011/303487

**Published:** 2011-07-13

**Authors:** Salem Alshemmari, Reem Ameen, Javid Gaziev

**Affiliations:** ^1^Department of Medicine, Faculty of Medicine, Kuwait; ^2^Department of Medical Laboratory Sciences, Faculty of Allied Health, Kuwait; ^3^International Center for Transplantation in Thalassemia and Sickle Cell Anemia, Mediterranean Institute of Hematology, Policlinico Tor Vergata, Viale Oxford 81, 00133 Rome, Italy

## Abstract

Haploidentical hematopoietic stem-cell transplantation is an alternative transplant strategy for patients without an HLA-matched donor. Still, only half of patients who might benefit from transplantation are able to find an HLA-matched related or unrelated donor. Haploidentical donor is readily available for many patients in need of immediate stem-cell transplantation. Historical experience with haploidentical stem-cell transplantation has been characterised by a high rejection rate, graft-versus-host disease, and transplant-related mortality. Important advances have been made in this field during the last 20 years. Many drawbacks of haploidentical transplants such as graft failure and significant GVHD have been overcome due to the development of new extensive T cell depletion methods with mega dose stem-cell administration. However, prolonged immune deficiency and an increased relapse rate remain unresolved problems of T cell depletion. New approaches such as partial ex vivo or in vivo alloreactive T cell depletion and posttransplant cell therapy will allow to improve immune reconstitution in haploidentical transplants. Results of unmanipulated stem-cell transplantation with using ATG and combined immunosuppression in mismatched/haploidentical transplant setting are promising. This paper focuses on recent advances in haploidentical hematopoietic stem-cell transplantation for hematologic malignancies.

## 1. Introduction

Hematopoietic stem-cell transplantation (HSCT) offers a curative treatment for many patients with malignant and nonmalignant hematologic disorders. As the probability of finding an HLA identical sibling donor is only 25% to 30%, attention has been focused on the use of alternative donors, either from unrelated adult donors, umbilical cord blood, or partially matched related donors. Currently, the chance of finding a matched unrelated donor varies from 60%–70% for Caucasians to less than 10% for ethnic minorities [1, 2]. Further drawbacks for a patient who urgently needs a stem-cell transplantation are the time interval from initiating an unrelated donor search to the identification of an appropriate donor of about 4 months [3], the considerable costs of high-resolution HLA typing, registry, and banking expenditures, and the absence of donor registries in many countries worldwide. Unrelated cord blood has become a new promising stem-cell source due to its faster availability, tolerance of 1-2 HLA mismatches out of 6, lower incidence and severity of acute graft-versus-host disease (GVHD), and lack of risk to the donor. However, the limited unit size, high incidence of posttransplant infections, and slow immune recovery are obstacles to its broader application especially in adult patients [4, 5]. Haploidentical related donor transplantation has been developed to address limitations in allogeneic transplant donor availability. Virtually all patients have a one haplotype-mismatched donor. In addition to immediate availability, haploidentical donors have several advantages such as (a) ability to select the best donor on the basis of age, sex, and infectious disease status, (b) optimal graft composition, and (c) prompt access to repeated donation in case of graft failure or need for cellular therapy after transplant. The initial studies of haploidentical HSCT employed myeloablative conditioning regimen followed by infusion of unmanipulated bone marrow grafts, and GVHD prophylaxis with methotrexate (MTX), with or without cyclosporine (CSA) [6–8]. Outcome of these transplants was poor due to high rate of graft failure, severe GVHD, and nonrelapse mortality (NRM), especially when donors were mismatched for 2 or more antigens. Over the past decade results of haploidentical HSCT have improved significantly due to novel graft manipulation techniques, improved prophylaxis of GVHD, and the development of new conditioning regimens. This paper focuses on recent developments which have led to more encouraging results in haploidentical HSCT for malignant diseases in adult patients.

## 2. Early Experience with Haploidentical Bone Marrow Transplantation

In 1983, Powles et al. [9] reported the outcome of 35 patients with acute myeloid or lymphoblastic leukaemia who received a 1–3 HLA-mismatched marrow grafts from parent, child, or sibling, following cyclophosphamide/total body irradiation or cyclophosphamide/melphalan conditioning regimens. There were 11 patients surviving at 6 months and only 5 patients more than 2 years. Graft failure occurred in 29% of patients and GVHD in 80% of patients. The poor survival rate with nonrelapse mortality occurring in more than half of the patients was discouraging. 

In the late 1980s, the Seattle group reported the results of HLA-mismatched/haploidentical transplantation using unmodified bone marrow and myeloablative conditioning in patients with haematological malignancies [6–8]. They demonstrated that the probability of survival for patients who underwent transplantation from an HLA 1-locus-mismatched family member was similar to that for patients who received a graft from an HLA-identical sibling, while results of transplantation from 2 or 3 loci-mismatched family member were extremely poor, owing to the high incidence of graft failure and severe GVHD. The results of these early studies were disappointing and highlighted the inherent difficulties of haploidentical transplantation using unmodified bone marrow and limited its application to many patients who could benefit from transplantation.

## 3. Haploidentical Transplantation with Partial Ex Vivo T-Cell Depletion Combined with Intensive Immunosuppression

Starting in the early 1990s, several investigators set to work on strategies to overcome HLA barriers and control GVHD in haploidentical transplantation through partial depletion of bone marrow combined with intensive immunosuppression [10–12]. In these studies, the incidence of grade 2–4 acute GVHD has ranged from 18% to 40% in patients of HLA-mismatched bone marrow transplantation after T-cell depletion using anti-CD6 or T_10_B_9_ monoclonal antibodies.

Henslee-Downey et al. [12] in their pioneering work used marrow grafts depleted with T_10_B_9_ monoclonal antibodies combined with intensive posttransplant immunosuppression with CSA, ATG, and methylprednisolone in 72 patients mainly affected by hematologic malignancies following TBI-based myeloablative conditioning. Thirteen patients received 1 antigen, 35 patients 2 antigen, and 24 patients 3 antigen mismatched grafts. The probability of initial engraftment was 88%, and it was 96% following second transplantation. Importantly, the incidence of grade 2–4 or 3-4 acute GVHD was 16% and 7%, respectively. Chronic extensive GVHD occurred in 4 of 48 (8%) evaluable patients. In all, the probability of 2 year survival was 35%, and it was 55% for low-risk patients. Relapse occurred in 32% of patients. 

Recently, investigators from the same institute have reported the outcome on 201 patients with acute leukaemia (67% not in remission) who received marrow grafts from partially mismatched related donors (92% mismatched for 2-3 HLA antigens) [13]. Conditioning regimen consisted of TBI, cyclophosphamide, cytarabine, etoposide, ATG, and methylprednisolone. GVHD prophylaxis comprised partial T-cell depletion with OK3 or T_10_B_9_, steroids, ATG and cyclosporine. Impressively, the cumulative incidence of grades 2–4 acute GVHD and chronic GVHD were 13% and 51%, respectively. However, the probabilities of OS and DFS at 5 years were low (19% and 18%, resp.). OS and DFS probabilities were better in patients transplanted in remission (34% and 29%, resp.). The cumulative incidence of relapse at 5 years was 31%. Transplant-related mortality was 51%. Patient age >15 years, active disease at transplant, donor age >25 years, and 3-antigen mismatches affected the outcome adversely, emphasizing the importance of patient selection and disease status for good outcome.

## 4. Haploidentical T-Cell Depleted Transplantation Using CD34+ Selection

The modern era of haploidentical transplantation via CD34+ selection started with clinical studies by the Perugia University transplant team from Italy. They developed a new approach to ex vivo T-cell depletion with a “mega dose” stem cells following myeloablative TBI-based conditioning regimen. The concept of mega dose of stem cells came from animal models, where higher doses of stem cells overcame major histocompatibility barrier (MHC), by presumed “veto effect” [14, 15]. 

In their pioneering work, Aversa et al. [16] transplanted mega doses of stem cells obtained from bone marrow and G-CSF-stimulated peripheral blood following T-cell depletion with soybean agglutination and E-rosetting technique, without any subsequent GVHD prophylaxis in 17 patients with end-stage leukemia. One patient had rejection, and all the remaining patients achieved sustained engraftment. One patient died from grade 2 acute GVHD, and no other patients developed grade ≥2 acute GVHD. Ruggeri et al. [17] from the same team showed that killer immunoglobulin-like receptor (KIR) mismatching in the GVH direction is associated with strikingly less relapse following transplantation for AML patients although such effect was not observed in ALL patients. The Perugia team subsequently adopted new depletion technique with the E-rosetting followed by CD34-positive selection using Ceprate [18], or positive selection of CD34 cells from peripheral blood by magnetic beads using the CliniMACS (Myltenyi) system [19]. The development of T-cell depletion method using CliniMACS system, by the investigators from Tuebingen, Germany, has revolutionized the field of haploidentical transplantation which provides a good yield of CD34+ cells with T-cell depletion of more than 4 logs and B-cell depletion of over 3 logs [20], thus also allowing to lower the incidence of EBV-associated lymphoproliferative disorders usually frequent in extensive T-cell deleted mismatched transplantation. Apart from effective T-cell depletion, this automated graft processing system provides reproducible CD34 yield and allows to perform T-cell-depleted haploidentical transplantation in any transplant center. 

In a phase II trial, the Perugia team has reported results of HLA-haploidentical transplants for adults with high risk acute myeloid (*n* = 67) or lymphoid leukemia (*n* = 37) [19]. Conditioning regimen consisted of 8 Gy TBI, thiotepa, fludarabine and ATG with no GVHD prophylaxis after transplantation. PBSC graft consisted of CD34+ cells selected using the CliniMACS one-step procedure in 88 donors and the Isolex two-step procedure (positive selection of CD34+ cells followed by negative selection of CD3+ cells) in 16 donors. Median CD34+ cell dose was 13.8 × 10^6^/kg, and it was 1 × 10^4^/kg for CD3+ cells. Ninety-four (91%) of 101 evaluable patients achieved primary engraftment. Rejection occurred in 7 (7%) patients. Acute grade 2 or grade 3-4 GVHD developed in 6 and 2 patients, respectively. Extensive chronic GVHD occurred in 3 patients. High TRM (40%) occurred mostly from infection was probably anticipated, given that many patients were heavily pretreated and had advanced disease status at transplantation. The cumulative incidence of relapse was 16% in patients transplanted in remission and 51% in those transplanted in relapse. Relapse was less frequent (14%) in AML patients receiving graft from NK-alloreactive donors then patients whose donor was not NK-alloreactive (28%) although the difference was not statistically significant. Two-year probability of EFS in 42 AML and 24 ALL patients receiving transplant in any complete remission was 48%  ± 8% and 46%  ± 10%, respectively. However, patients transplanted in relapse had only 4% of EFS probability. 

In a recent multicenter registry-based study, Ciceri et al. [21] reported on 266 adult patients with de novo acute leukemia who received haploidentical transplantation. The conditioning regimen was similar to the Perugia protocol. This study confirmed that a high engraftment rate and minimal GVHD in the absence of any posttransplant immunosuppression after HSCT from haploidentical donors can be achieved by infusion a large number of CD34+ selected cells. Leukemia-free survival at 2 years was 48%  ±  10%, 21%  ± 5%, and 1% for patients with AML who received transplantation in CR1, ≥CR2, and nonremission. In ALL patients, the same probabilities were 13%  ±  7%, 30%  ±  8%, and 7%  ±  5%, respectively. As in the Perugia experience, TRM, mostly from infection, was observed in a significant proportion of patients indicating that fast immune recovery is essential for successful transplant outcome.

In a Canadian multicenter study [22], the outcome of haploidentical transplantation in 11 patients with AML who received CD34+ selected grafts following a modified Perugia protocol was reported. All patients had sustained engraftment, and none developed GVHD. However, death from relapse or infection occurred in 10 of the 11 patients highlighting that delayed immune reconstitution leading to high infection-related mortality remains a major obstacle in haploidentical transplants. 

Kato et al. [23] from Japan have reported a nationwide survey of the outcome of haploidentical transplantation on 135 patients following CD34+ selected from bone marrow or GCSF-mobilized PBSC grafts using the Isolex device. Twenty-eight donors were mismatched for 0-1 antigen, 64 for 2, and 43 for 3 antigens. Thirty-eight patients received CD34+ selected cells from bone marrow, 74 patients from PBSC, and 23 patients from both. TBI-based conditioning regimen was used in 104 of 110 patients with malignancy. ATG was given to 47 patients. Posttransplant GVHD prophylaxis with CSA, tacrolimus, MTX, or steroids either alone or in combination was given to 105 patients. The median CD34+ cell dose was lower than that used the Perugia group: 3.2 × 10^6^/kg, for patients receiving bone marrow, 5.5 × 10^6^/kg for patients receiving PBSC, and 4.9 × 10^6^/kg for those receiving both. The median CD3+ cell dose was: 6.0 × 10^4^, 9.4 × 10^4^, and 12.1 × 10^4^ for BM, PBSC, and BM + PBSC groups, respectively. Graft failure occurred in 13% of patients with malignancies and 40% in nonmalignant diseases. Importantly, the incidence of grade 3 acute GVHD was low 8.4%, and no patient developed grade 4 acute GVHD. Disease-free survival at 5 years was 39% in standard risk and 5% in high risk patients, similar to those observed in other studies of haploidentical transplantation. The results of this study show that standard doses of CD34+ cells probably are not sufficient for sustained engraftment in nonmalignant diseases and posttransplant immunosuppression could be necessary to prevent severe GVHD in haploidentical transplants with moderate degree of T-cell depletion.

## 5. Haploidentical Transplantation Using CD3/CD19 Depletion

Investigators from Tuebingen, Germany, used mega dose of positive CD34+ selected grafts from HLA-mismatched parental donors following myeloablative conditioning regimen which resulted in high transplant-related mortality [24]. Consequently, they adopted a new regimen using graft CD3/CD19-depleted with anti-CD3-and anti-CD19-coated microbeads on a CliniMACS device. Such grafts not only contain CD34+ stem cells but also CD34 negative progenitors, natural killer cells, and dendritic and graft-facilitating cells which could enhance engraftment and fast immune recovery after transplantation. The use of CD3/CD19 depleted grafts following a reduced intensity conditioning regimen was proposed as an alternative to overcome negative consequences of myeloablative conditioning regimen with CD34+ positive selection. 

Lang et al. [25] in a pilot study of 11 children with hematologic malignancies used CD3/CD19 depleted grafts following a reduced intensity conditioning (RIC) regimen consisting of fludarabine, melphalan, and thiotepa; primary engraftment rate was 91% with faster CD3+ T-cells reconstitution as compared with patients who received CD34+ and CD133+ selected grafts. 

Recently, Bethge et al. [26] reported results of haploidentical transplantation on 29 adult patients with hematologic malignancies (AML = 16, ALL = 7, NHL = 3, MM = 2, CML = 1) who received CD3/CD19 depleted grafts following a RIC regimen consisting of fludarabine, thiotepa, melphalan, and OKT-3. Fifteen patients had relapse following previous allogeneic or autologous transplantation, and the remaining patients had refractory disease. Posttransplant immunosuppression with mycophenolate mofetil was used if CD3+ cell dose in the graft exceeded 5 × 10^4^/kg. The CD3/CD19 depleted grafts contained a median of 7.6 × 10^6^/kg CD34+ cells/kg, 4.4 × 10^4^ CD3+ cells/kg, and 7.2 × 10^7^ CD56+ cells/kg. All but one patient engrafted. Grade 2–4 GVHD occurred in 48% of patients. Twenty of 29 patients (68%) died, 12 from relapse, 7 from infection, and 1 from GVHD. The probability of EFS at 12 months was 35%. Results of this study demonstrate that HSCT with CD3/CD19 depleted grafts without mega dose of CD34+ cells is feasible, safe and resulted in an acceptable survival rate in heavily pretreated higher risk patients. 

In a prospective multicenter phase II study of haploidentical HSCT in 60 adults (including patients reported in the previous study) with hematologic malignancies using RIC and CD3/CD19 depleted grafts Ferdermann et al. [27] reported clinical outcomes and immune reconstitution. Patients were treated with the same conditioning regimen as reported by Bethge et al. [26]. At transplantation 30 patients were in CR and 30 in partial remission. Grafts contained median of 6.8 × 10^6^ (range 3.5–22) CD34+ cells/kg, 4 × 10^4^ (range, 0.9–44) CD3+ cells/kg and 2.8 × 10^7^ (range 0.00–37.3) CD56+ cells/kg. The incidence of grade 2–4 acute GVHD and chronic GVHD was 47% and 15%, respectively. The probability of survival at 1 and 2 years were 41% and 24%, respectively. Overall nonrelapse was high 44%. There was no positive impact of KIR-mismatch on survival even in the subgroup of patients with AML. Recovery of NK cells was early and fast. Although T- and B-cell reconstitution was delayed, it seemed faster compared with CD34+ selected transplants.

Recently this group has reported results of immune reconstitution after haploidentical HSCT using RIC and CD3/CD19 depleted grafts in 28 patients [28]. Engraftment was rapid and sustained without mega doses of CD34+ cells with full chimerism reached on day +15 after transplantation. Large amount of NK cells resulted in fast NK-cell recovery. T- and-B-cell reconstitution was delayed but was faster than reported after CD34+ selection and myeloablative conditioning. These data show that despite relatively fast immune reconstitution following CD3/CD19-depleted grafts nonrelapse mortality remains still high at least in adult patients.

## 6. Posttransplant High-Dose Cyclophosphamide for Selective In Vivo T-Cell Depletion

It has been shown in animal models that both graft rejection and GVHD after histoincompatible BMT can be inhibited by the posttransplant administration of high-dose cyclophosphamide, which is known to be highly toxic to lymphocytes proliferating in response to recent antigen stimulation [29]. Based on this experience, investigators from Johns Hopkins first demonstrated the feasibility of unmanipulated bone marrow transplantation from haploidentical related donors after nonmyeloablative conditioning regimen, followed by posttransplant high-dose cyclophosphamide in thirteen patients with high risk malignancies [30]. Conditioning regimen consisted of fludarabine and low-dose TBI in 3 patients (cohort 1), while the remaining 10 patients also received cyclophosphamide 29 mg/kg in two days (cohort 2). All patients received cyclophosphamide 50 mg/kg on day 3 and mycophenolate mofetil and tacrolimus from day 4 onward after transplantation. Two patients (both in cohort 1) rejected their grafts; grade 2 or 3 acute GVHD occurred in 6 patients. Six out of 10 patients in cohort 2 were alive, and 5 of them in complete remission. Updating their experience, Luznik et al. [31] reported transplant outcomes of 68 patients who received a haploidentical nonmyeloablative bone marrow transplant for hematologic malignancies using high-dose posttransplant cyclophosphamide at Johns Hopkins or Fred Hutchinson Cancer Research Center. Nonmyeloablative conditioning regimen consisted of fludarabine, cyclophosphamide, and TBI. As GVHD prophylaxis, they received posttransplant cyclophosphamide on day 3 or days 3 and 4 and tacrolimus and mycophenolate mofetil starting day 5 after transplantation. Low incidence of acute GVHD (grades 2–4, 34% and grades 3-4, 6%) and nonrelapse mortality at 1 year (15%) with this new approach are very encouraging although there were a relatively high incidence of graft rejection (13%) and relapse rate at 1 year (51%).

## 7. Myeloablative Transplantation Using In Vivo T-Cell Depletion

The first study of haploidentical transplantation from mother to child in advanced leukaemia using busulfan/cyclophosphamide conditioning along with in vivo T-cell depletion with antilymphocyte globulin given before and after unmanipulated bone marrow transplantation was reported by the Pesaro group, Italy, in 1995 [32]. The probability of event-free survival and relapse was 26% and 33%, respectively. Severe acute GVHD occurred in 32% of patients. Although results were similar to those obtained in advanced leukaemia after HLA identical sibling transplants at the same institution, the data showed that rejection and severe GVHD remained high despite post transplant serotherapy.

Recently Lu et al. [33] from China reported results of HLA mismatched/haploidentical transplantation using G-CSF- primed bone marrow and peripheral blood with intensive immunosuppression including antithymocyte globulin (ATG) for in vivo T-cell depletion. Conditioning regimen consisted of cytosine arabinoside, busulfan, cyclophosphamide, and semustine, while GVHD prophylaxis included CSA, mycophenolate mofetil, and a short-course of MTX. In this study, they compared the clinical outcomes in HLA-haploidentical transplantation to HLA-identical sibling transplantation without ATG. The cumulative incidence of grades 2–4 GVHD in the matched and mismatched cohorts were 32% versus 40%  (*p* = NS), respectively. The incidence of chronic GVHD was similar in both groups. Surprisingly, treatment-related mortality and relapse rate were similar (14% versus 22% and 13% versus 18%, resp.) in both cohorts of patients. Updating the observations of this group to 250 patients [34] who underwent HLA-mismatched/haploidentical transplantation, the 3-year probability of leukemia-free survival in standard and high-risk acute myeloid leukemia was 70.7% and 55.9%, and in acute lymphoblastic leukemia, the respective probabilities were 59.7% and 24.8%. The incidence of acute GVHD grades 2–4 was 45.8%, and it was 13.4% for grade 3-4 GVHD. Although the overall incidence of chronic GVHD was 42.4%, its extensive form was only 14.3%. In this study, the low incidence of severe acute and chronic GVHD among the mismatched cohort is impressive and in part may be related to the use of ATG providing in vivo depletion of T-cells and combined posttransplant immunosuppression. 

Another approach to in vivo depletion is the use of alemtuzumab, a humanized monoclonal antibody that targets CD52 antigen that is expressed on many T and B cells as well as on some dendritic and NK cells. Kanda et al. [35] reported the clinical outcomes of haploidentical transplants in six patients treated with alemtuzumab combined with conventional TBI and cyclophosphamide and GVHD prophylaxis with CSA + short MTX for patients aged <50 years old. All six patients achieved engraftment, and only one patient developed acute GVHD, and 2 patients died from disease recurrence.

## 8. Reduced Intensity Transplantation Using In Vivo T-Cell Depletion

Historically transplant-related mortality was high in patients receiving mismatched transplantation following myeloablative conditioning. Therefore, several transplant teams have explored reduced intensity transplants in this setting.

Rizzieri et al. [36] reported outcomes of reduced intensity haploidentical transplants for 49 patients who received fludarabine, cyclophosphamide, and alemtuzumab as conditioning regimen and mycophenolate mofetil with or without CSA for GVHD prophylaxis. Importantly, the incidence of grade 2–4 or 3-4 acute GVHD was 16% and 8%, respectively, and treatment related mortality was 10.2%. Primary or secondary graft failure occurred in 6% and 8% of patients, respectively. The 1 year survival was 31% for all patients and 63% for patients in standard risk group. 

Recently, Ogawa et al. [37] from Osaka University, Japan, reported outcomes of haploidentical transplants in 26 patients with high-risk hematologic malignancies following reduced intensity transplantation of PBSC. Conditioning regimen consisted of ATG, fludarabine, busulfan, and GVHD prophylaxis consisted of tacrolimus and methylprednisolone. There was one rejection, and the incidence of grade 2–4 acute GVHD was only 19%. Four patients (15%) died of treatment-related complications, and six (23%) of progressive disease. Sixteen patients (62%) were alive in complete remission. These data are encouraging in terms of survival and transplant-related mortality although limited to small number of patients. 

In a recent study from Japan Kurokawa et al. [38] have reported on 66 patients treated with reduced intensity conditioning regimen consisting of TBI or fludarabine, ATG, busulfan, and melphalan. GVHD prophylaxis included tacrolimus ± methylprednisolone. Fifty patients received PBSC and twelve patients bone marrow. Four patients (6.1%) had rejection. Twenty three (38%) out of 60 evaluable patients developed acute grade 2–4 GVHD. Seven patients (12%) had relapse. Transplant-related mortality at 2 months was high 70%. Organ failure and infections were the main causes of mortality in 38 out of 46 patients. Overall survival was much worse for 28 patients who received HLA 3 loci mismatched grafts than for 11 patients receiving 1 locus mismatched grafts (12.5% versus 63.6%, resp.). Results of haploidentical transplants in this study were unsatisfactory as compared with previous study from the same country. Probably low dose of ATG, its timing, and the use of melphalan in the conditioning are responsible for inferior results as compared with the previous study by Ogawa et al. [37].

## 9. Haploidentical Transplantation after Ex Vivo Induction of Anergy

Although nonselective T-cell depletion combined with high CD34+ cell doses has been effective in ensuring a high rate of sustained engraftment and preventing severe GVHD in haploidentical transplants, the success of this strategy is limited by increased infectious complications, high relapse rate associated with delayed immune reconstitution, and loss of pathogen- and tumor-specific T-cells. Therefore, various experimental strategies to selectively deplete alloreactive T-cells within the graft to prevent GVHD while preserving pathogen- and tumor-specific immunity have been developed in an attempt to improve immune reconstitution after HLA-mismatched transplantations [12, 13]. Induction of allospecific anergy in donor T-cells before HSCT is an alternative to selective depletion. Activation of T-cells occurs through the engagement of both T-cell receptor and CD28 on the surface of T-cells by the MHC peptide and B7 family molecules on antigen presenting cells (APCs). Blockade of T-cell receptor signalling by fusion proteins (CTLA4-Ig) or monoclonal antibodies (anti-B7) that bind to the CD28 ligands B7 on APC lead to an anergy state [39]. In a recent study, Davies et al. [40] reported on 24 patients with hematologic malignancy (*n* = 21) or bone marrow failure syndromes (*n* = 3) who received haploidentical transplants by inducing T-cell anergy with CTL4-Ig or anti B7.1 and B7.2 antibodies. Patients received TBI-based conditioning regimen and CSA and short MTX as GVHD prophylaxis. Two patients had rejection, and two other patients died early before engraftment occurred. Only 5 (24%) of 21 evaluable patients developed severe GVHD. The incidence of chronic GVHD was low 8%. Twelve (50%) patients died of TRM. Actuarial EFS at 10 years was 33%. Importantly, the cumulative incidence of relapse/progression was low 17% although 14 patients had progressive disease at the time of transplantation indicating preserved graft-versus-tumor effect following such transplantation. Patients had rapid immune reconstitution, and there was no mortality from viral infections. Although high early TRMsv is mainly due to sepsis and multiorgan failure, results of this study are promising, suggesting that alloanergized BMT could be an alternative to nonselective T-cell-depleted haploidentical transplants especially in hematologic malignancies.

## 10. Major Complications of Haploidentical Transplantation

Recent advances with effective T-cell depletion and intensive conditioning regimen have significantly reduced the incidence of both GVHD and rejection after haploidentical transplantation for hematologic malignancies. Furthermore, development of reduced intensity conditioning regimen in haploidentical transplants resulted in reducing early transplant-related mortality and GVHD, while enabling sustained engraftment, and hence allowing for medically less-fit patients to benefit from haploidentical transplantation. However, posttransplant infectious complications and relapse remain the most important barriers yet to be overcome in this setting. Effective T-cell depletion in adults is associated with a profound and prolonged immunodeficiency that predisposes patients to life-threatening infections and, in the absence of donor lymphocyte mediated graft-versus tumor effect, to leukaemia relapse, both representing leading cause of death in this treatment approach. 

In a recent study by Aversa et al. [19], nonrelapse mortality occurred in 28 (36.5%) patients and 27 of them died from infections with cytomegalovirus as the leading cause (52%) followed by fungal infections (19%). Although the incidence of adenovirus infection is low in adult patients than in children, it is still a significant cause of nonrelapse mortality in haploidentical transplants. Epstein-Barr virus reactivation is a frequent event after T-cell depleted stem-cell transplantation due to the lack of donor-derived EBV-specific cytotoxic T-cells in the early period after transplantation which is a risk factor for the development of lymphoproliferative disorders. Intensive screening for these viruses in pre- and posttransplant period and timely use of preemptive antiviral agents (ganciclovir or foscarnet for CMV and cidofovir for adenovirus) or monoclonal antibodies (rituximab for EBV) can prevent progression in disease in most patients. However, in profound immunodeficient patients, even with preemptive therapy, it is difficult to obtain long-lasting control of infection/disease. Therefore, approaches such as adoptive transfer of donor-derived CMV, adenovirus or EBV-specific T-cells may help to protective immunity in the early post transplant phase, and hence reduce nonrelapse mortality in haploidentical transplants. Over the past decade there has been considerable progress in generating nonalloreactive T-cells specific against different pathogens as CMV, adenovirus, EBV and aspergillus species. Leen et al. [41] have evaluated efficacy of generated antigen specific cytotoxic T lymphocytes (CTL) against CMV, adenovirus and EBV in immunocompromised recipients. Dose range of CTL was 5 × 10^6^ to 1.35 × 10^8^/m^2^. Twelve patients received bivirus and 15 patients trivirus CTL. No dose limiting toxicity was observed. Most patients showed decreasing or clearance of viral load. Although very promising results, these approaches require high resources and are characterized by specific intrinsic limitations that prevent a widespread use.

Leukemia relapse is another common complication occurring in haploidentical T-cell depleted transplants. The cumulative incidence of relapse was 16% in patients transplanted in remission and 51% in those transplanted in relapse [19]. Eight patients were given rescue therapy for relapse (DLI in 5, second transplant in 2, and DLI/imatinib in one patient), while in 18 patients rapid progression of disease precluded any rescue treatment. The only ALL/Ph+ patient treated with DLI/imatinib was alive in remission. Dismal outcome after haploidentical T-cell depleted transplants in relapse indicates that for these patients alternative strategies should be considered.

## 11. Approaches to Overcome Drawbacks of Haploidentical T-Cell-Depleted Transplantation

To overcome limitations of haploidentical T-cell depleted transplantation, hence enabling physician to perform safe and more successful transplants, much efforts have been made in selecting the best donor and/or developing immunological modulation after transplantation.

### 11.1. Role of NIMA-Mismatched Haploidentical Transplants

Bidirectional fetomaternal hematopoietic cell trafficking during pregnancy can lead to long-lasting microchimerism which can have a beneficial effects on transplantation outcome by reducing host-versus-graft or graft-versus-host alloreactivity not only against noninherited maternal antigens (NIMA) but also against noninherited paternal antigens (NIPA) in the setting of stem-cell transplantation [42, 43]. In a large IBMTR analysis, van Rood et al. [44] first showed a low rate of acute GVHD and TRM in sibling who received T-cell replete bone marrow transplantation mismatched for NIMAs compared with those mismatched for NIPAs, suggesting the presence of immunological tolerance against NIMAs in haploidentical transplants. Several studies from Japan have confirmed the tolerizing effect of NIMAs [45, 46]. However, despite relatively low incidence of acute GVHD in NIMAs-mismatched transplants, the incidence of chronic extensive GVHD is still high in such T-cell replete approach [47] indicating that presumed immunologic tolerance alone is not sufficient to significantly reduce GVHD and improve transplant outcomes.

### 11.2. Role of NK Cell Alloreactivity and NK Cell Infusion in Haploidentical Transplants

It has been suggested that NK cell alloreactivity is a powerful tool against leukemic cells, allowing an effective antileukemic activity following haploidentical HSCT, especially in AML patients. KIR-ligand incompatibility in GVH direction has been associated with lower relapse rate and improved leukemia-free survival in adult patients with AML who received transplant in remission [48]. However, patients who are not in remission have a substantially high risk of relapse or progression, probably due to an immune escape from the selective pressure of donor T-cells owing to the loss of the patient-specific HLA haplotypes with the frequency of 30% [49]. As KIR-ligand mismatch could exert antirelapse activity, several groups have evaluated the feasibility of infusing NK cells in haploidentical transplants. Recently, investigators from Duke University, USA, have reported the feasibility of NK cell infusion in 14 HLA matched and 16 mismatched nonmyeloablative transplanted patients [50]. Single-step NK cell enrichment by CD56+ selecting column through ClinMACS system yielded approximately 90% of NK cells of interest, with a low level of CD4 and CD8 cells responsible for GVHD development. In the mismatched patients, the median dose of CD3−CD56+ cells /kg and contaminating CD3+CD56+ cells infused were 9.21 × 10^6^/kg and 0.27 × 10^6^, respectively, without significant toxicity. Only 1 patient in the mismatched setting developed grade ≥3-4 acute GVHD. Results of this study showed that enriched NK cells infusion is safe and has the potential to improve immune recovery and outcomes although it should be demonstrated in randomized studies.

### 11.3. Role of DLI in Haploidentical Transplants

Posttransplant donor lymphocyte infusion (DLI) provided direct and potent GVL activity to treat relapse in patients receiving HLA matched both related and unrelated transplants. Limited number of patients who received haploidentical transplant given unmodified DLI, both prophylactically and therapeutically, does not permit to draw definite conclusion about the relationship between cell dose, GVHD, and GVL effect. However, the use of unmodified DLI can cause lethal GVHD. Various approaches have been developed to circumvent this, including induction of anergy [39], add back of regulatory T-cells [51], and transduction of donor lymphocytes with suicide genes [52, 53]. 

Recently, Ciceri et al. [54] reported results of a non-randomised phase I-II study on the use of suicide-gene-engineered donor lymphocytes after haploidentical HSCT for 28 patients with leukaemia. Patients were given escalating doses of donor lymphocytes expressing herpes-simplex thymidine kinase suicide gene (TK-cells) starting at 28 days after transplantation. Of the 28 patients infused, 22 (88%) showed TK-cell engraftment who achieved CD3+ counts of 100 cells/*μ*L or more between 13 and 42 days from infusion. Interestingly, the 6 patients without engraftment remained immunodeficient. Ten (45%) of 22 immune-reconstituted patients developed grade 1–4 acute GVHD, and one developed extensive chronic GVHD. Ten patients needed ganciclovir treatment which resulted in a significant reduction of circulating TK-cells. No patient died from GVHD. Patients showed normalization of cytomegalovirus and Epstein-Barr virus- (EBV-) specific T-cell recovery within 3 months of transplantation. Remarkably, in immune reconstituted patients, progressive normalisation of antiviral responses was associated with decline in the number of infectious events as compared with patients who failed immune reconstitution. Consequently, the cumulative incidence of nonrelapse mortality at 100 days was low, 14% (with infectious mortality of 9%) in TK-treated immune-reconstituted patients versus 60% in nonimmune-reconstituted patients. The authors of this study concluded that a good survival rate of 49% observed in patients with de novo leukemias might be due to the absence of post transplant immunosuppression and early add back of TK-cells resulted in a reduced infectious mortality. Results of this study showed feasibility and benefit from early TK-suicide-gene induced lymphocytes infusion which resulted in fast immune reconstitution and decreased nonrelapse mortality due to infection.

Amrolia et al. [55] used an anti-CD25 immunotoxin to deplete alloreactive lymphocytes and infused allodepleted donor T-cells after ex vivo T-cell depleted haploidentical transplants. They demonstrated that viral-specific responses were observed in 4 of 6 evaluable patients receiving higher doses of T-cells, with only two patients developing significant acute or chronic GVHD. Results of this study are very promising in terms of prevention of sever GVHD, but this benefit was offset by high leukaemia relapse (43%).

### 11.4. Role of T Regulatory Lymphocytes in Haploidentical Transplantation

Hoffmann et al. [56] showed that CD4+CD25+ T-cells (Tregs) suppress GVHD in a murine model of allogeneic BMT across a complete MHC class I and II barrier. In a murine GVHD model, adoptive transfer of Tregs resulted in abrogation of GVHD and enhancement of immune reconstitution, hence protecting animals from lethal CMV infection [57]. Recently, researchers from the Perugia team have reported outcomes of haploidentical transplantation in 28 patients with hematologic malignancies (AML = 22, ALL = 5, NHL = 1) who received CD34+ selected grafts and Tregs of infusion [58]. After conditioning with TBI, thiotepa, fludarabine, and cyclophosphamide patients received fresh donor Tregs isolated by immunoselection on day-4, and CD34+ selected cells (mean 9.4 × 10^6^  ±  3.4/kg) and conventional (Tcons) lymphocyte infusion on day 0. No posttransplant immunosuppression was given. The first 4 patients received 0.5 × 10^6^/kg of Tcons and 2 × 10^6^/kg of Tregs. The absence of GVHD allowed them to escalate Tcons to 1 × 10^6^/kg in the next 17 patients, while the dose of Tregs remained unchanged. The last 5 patients received Tcons 2 × 10^6^/kg and Tregs 4 × 10^6^/kg. Two patients did not receive Tcons, because FoxP3+ expression in the Treg preparation was <50%. Twenty four patients had sustained engraftment. Two of 26 evaluable patients developed grade 2–4 acute GVHD and none chronic GVHD. Both patients received Tcons of 2 × 10^6^/kg. Patients had rapid increase in peripheral lymphocytes with 100 *μ*L of CD4 and CD8 cells between 28–135 and 19–95 days, respectively. There was early emergence of specific CD4+ and CD8+ for pathogens as aspergillus, candida, cytomegalovirus, adenovirus, herpes simplex virus, varicella zoster virus, and toxoplasma as compared with previous patients who received standard haploidentical transplant. Thirteen patients died, 8 of them from infectious complications although 4 patients had pretransplant fungal infection. Only one patient had relapse. Results of this study are very interesting and promising. Fewer incidence of GVHD despite high doses of early unmodified donor lymphocyte infusion is impressive, indicating that donor Tregs are able to abrogate GVHD and faster early immune recovery which is a key problem in haploidentical transplantation.

### 11.5. Role of Photodepletion in Haploidentical Transplants

Photodepletion of alloreactive T-cells is another approach to overcome drawbacks of haploidentical T-cell depleted transplants. Roy et al. [59] have reported on 19 patients who received ex vivo T-cell depleted haploidentical transplantation followed by administration of donor lymphocytes photodepleted of alloreactive T-cells. None of the 12 patients receiving high CD3+ cell doses (3.2 × 10^5^ to 5.0 × 10^6^/kg) developed grade 2–4 GVHD, and 2-year survival rate was 82%.

## 12. Conclusion

Haploidentical transplants offer a potential cure for patients in need of allogeneic transplantation when an HLA-matched either related or unrelated donor is not available. It is a reasonable option for patients who do need urgent transplantation or also for non-Caucasian patients, in whom a chance of finding an unrelated matched donor is still low. Recent advances with effective T-cell depletion have made it possible to perform haploidentical transplantation with a high sustained engraftment rate, lower early post transplant mortality and low rates of severe GVHD. However, poor immune recovery with associated infectious complications leading to late mortality, and relapse remain major obstacles to overcome. Donor selection based on NIMA matching or KIR typing and novel posttransplant cellular therapies will allow to further improving transplant outcomes in haploidentical transplants. Selective ex vivo or in vivo T-cell depletion methods appear promising and might be effective in preserving tumor- and pathogen-specific immunity while eliminating alloreactive T-cells responsible for GVHD. The data presented in this paper demonstrate important achievements made during the last decade in the field of haploidentical transplantation and great interest of scientific community in promoting the field with the ultimate goal being to cure more patients in need of stem-cell transplantation.
